# Features that matter: Evolutionary signatures can predict viral transmission routes

**DOI:** 10.1371/journal.ppat.1012629

**Published:** 2024-10-21

**Authors:** Maya Wardeh, Jack Pilgrim, Melody Hui, Aurelia Kotsiri, Matthew Baylis, Marcus S. C. Blagrove

**Affiliations:** 1 Department of Computer Science, University of Liverpool, Liverpool, United Kingdom; 2 Institute of Infection, Veterinary and Ecological Sciences, University of Liverpool, Liverpool, United Kingdom; American University of Iraq Baghdad, IRAQ

## Abstract

Routes of virus transmission between hosts are key to understanding viral epidemiology. Different routes have large effects on viral ecology, and likelihood and rate of transmission; for example, respiratory and vector-borne viruses together encompass the majority of rapid outbreaks and high-consequence animal and plant epidemics. However, determining the specific transmission route(s) can take months to years, delaying mitigation efforts. Here, we identify the viral features and evolutionary signatures which are predictive of viral transmission routes and use them to predict potential routes for fully-sequenced viruses in silico and rapidly, for both viruses with no observed routes, as well as viruses with missing routes. This was achieved by compiling a dataset of 24,953 virus-host associations with 81 defined transmission routes, constructing a hierarchy of virus transmission encompassing those routes and 42 higher-order modes, and engineering 446 predictive features from three complementary perspectives. We integrated those data and features to train 98 independent ensembles of LightGBM classifiers. We found that all features contributed to the prediction for at least one of the routes and/or modes of transmission, demonstrating the utility of our broad multi-perspective approach. Our framework achieved ROC-AUC = 0.991, and F1-score = 0.855 across all included transmission routes and modes, and was able to achieve high levels of predictive performance for high-consequence respiratory (ROC-AUC = 0.990, and F1-score = 0.864) and vector-borne transmission (ROC-AUC = 0.997, and F1-score = 0.921). Our framework ranks the viral features in order of their contribution to prediction, per transmission route, and hence identifies the genomic evolutionary signatures associated with each route. Together with the more matured field of viral host-range prediction, our predictive framework could: provide early insights into the potential for, and pattern of viral spread; facilitate rapid response with appropriate measures; and significantly triage the time-consuming investigations to confirm the likely routes of transmission.

## Author summary

Routes of virus transmission–the mechanism(s) by which a virus physically gets from an infected to an uninfected host, are crucial to understanding how viral diseases spread among animals and plants. Here, we uncover the evolutionary signatures which can predict the transmission routes a virus uses to move from one host to another, enabling us to identify any unobserved routes for known viruses and even predict potential routes of newly emerged viruses. We first compile a comprehensive dataset of virus-host associations. Using this dataset, we employ a multi-perspective machine learning approach to achieve high predictive performance. Our framework ranks viral features by their significance in prediction, revealing genomic evolutionary signatures linked to each route. Our approach could provide early insights into viral spread patterns, facilitating prompt response efforts to new outbreaks and epidemics, and streamline laboratory investigations. Overall, our study represents a step forward in our ability to anticipate and mitigate the impact of emerging infectious diseases on human, animal, and plant health.

## Introduction

Mounting an effective response to an emerging virus requires establishing all critical information, as quickly as possible. In recent years, significant focus has been placed on understanding determinants of host range and potential for spillover [1–5]. However, the transmission route, i.e. the pathway a virus uses to physically get from an infected to an uninfected host, still entails months, or even years, to thoroughly investigate. This was most apparent during the initial phases of the SARS-CoV-2 pandemic, where the relative importance of lingering aerosols versus fomite transmission was still being determined [6,7]. Furthermore, secondary transmission routes, such as sexual transmission of both Zika [8] and Ebola [9] viruses, are often only ascertained during significant outbreaks. The ability to identify all epidemiologically significant transmission routes of a virus, with high accuracy, computationally, with minimal information, and as quickly as possible, is therefore of paramount importance to the mitigation of future emerging viruses.

The transmission routes of a virus are also fundamentally intertwined with its ecology, epidemiology [10], and its potential for host shifting and spillover [11]. they determine how the virus spreads within and between different host populations, therefore influencing the potential, severity, and geographical extent of outbreaks. In animals, transmission routes such as respiratory, via droplets or aerosol, can result in rapid virus spread through a dense population. Influenza A, many coronaviruses, and the more benign rhinoviruses, all benefit from this transmission mechanism to cause swift outbreaks worldwide [12]. Conversely, vector-borne viruses tend toward a more varied outbreak speed closely linked with environmental temperature. For instance, the gradual spread of Usutu across temperate Europe [13], compared with the El Niño-driven rapid spread of Zika through South America [14]. But nonetheless, vector-borne routes can produce wide ranging and long-term establishment, for example dengue [15], bluetongue [16], and maize chlorotic mottle [17] viruses. Different epidemiological patterns are also seen for other sets of transmission routes, such as vertical, sexual, and water-borne [10,18].

In the plant kingdom, most viruses are transmitted by vectors, particularly by hemipterans insects, such as aphids and whiteflies [19]. The dynamics of this transmission by the vector: non-persistent, semi-persistent, or persistent, determine the length of window to disseminate the virus to a new plant after feeding (seconds to minutes, hours to days, or days to weeks, respectively) [20]. Vertical transmission via seeds, on the other hand, enables the virus to persist for considerably long periods when hosts or vectors are not available [21], and may allow it to disseminate over long distances, including continental jumps [22].

To facilitate computational prediction of transmission routes, we first compiled a dataset of known transmission routes of the animal and plant viromes, to their hosts, and used it to construct a hierarchy of transmission mechanisms. We then established a field-bridging and uniform methodology to define routes of transmission based on each virus-host association rather than a ‘per virus’ definition, because, in some cases, the same species or strain of virus may utilise a different range of transmission routes to infect different hosts. For instance, Influenza A is faecal-orally transmitted in waterfowl [23], but undergoes respiratory transmission in humans [24]. In other cases, very closely related, but different, viruses may utilise a diverse set of transmission routes in different hosts. For example, whilst many viruses in the family Flaviviridae are exclusively vector-borne, some are also vertically or sexually transmitted in both vertebrate and vector populations [25], further some, e.g. Hepatitis C virus, are blood-borne and do not replicate in arthropods [26].

Given these (common) complex examples of a virus using different routes for different hosts, and two closely related viruses using very different routes, we incorporated pair-wise association-level similarities into a unified framework, termed: ‘Virus-host integrated neighbourhoods’, to synthesise complementary predictive features. Furthermore, in order to enable parameterisation of transmission routes that are closely interlinked with host taxonomy (e.g. seed and pollen-borne routes are strictly limited to plants), we incorporated similarity between hosts to differentiate between those categorically different routes.

Finally, as virus structure has been shown to constrain virus transmission [20,27], and biases in the virus genome composition (e.g. stability, codon bias, etc.) can also inform the transmission mechanism deployed by the virus, as well as correlating with virus reservoirs and vectors [28], we synthesised a complementary array of variables from the full genome sequences of viruses.

We combined the above features and viral evolutionary signatures into lightGBM ensembles. We used those ensembles to identify which of our features are most predictive of transmission routes deployed by animal- and plant-infecting viruses to their known hosts; to predict which of those mechanisms are applicable to virus-host associations without observed transmission routes; and to establish potential gaps in our current knowledge of the transmission routes of known viruses to their animal or plant hosts. We present the full range of features which are predictive of each transmission route and discuss the mechanisms and contribution of the major viral evolutionary signatures correlated to the transmission route.

This study is the most taxonomically broad study of its kind, to demonstrate the potential of sequence and morphological information, increasingly available within the first few days of an outbreak, to predict the transmission routes of animal and plant viruses. Deployment of our framework could provide early insights into the potential for, and pattern of spread of a virus; facilitate rapid response with appropriate measures; and significantly triage the time-consuming investigations to confirm the likely routes.

## Results

### Hierarchy of virus transmission

**Fig 1:**
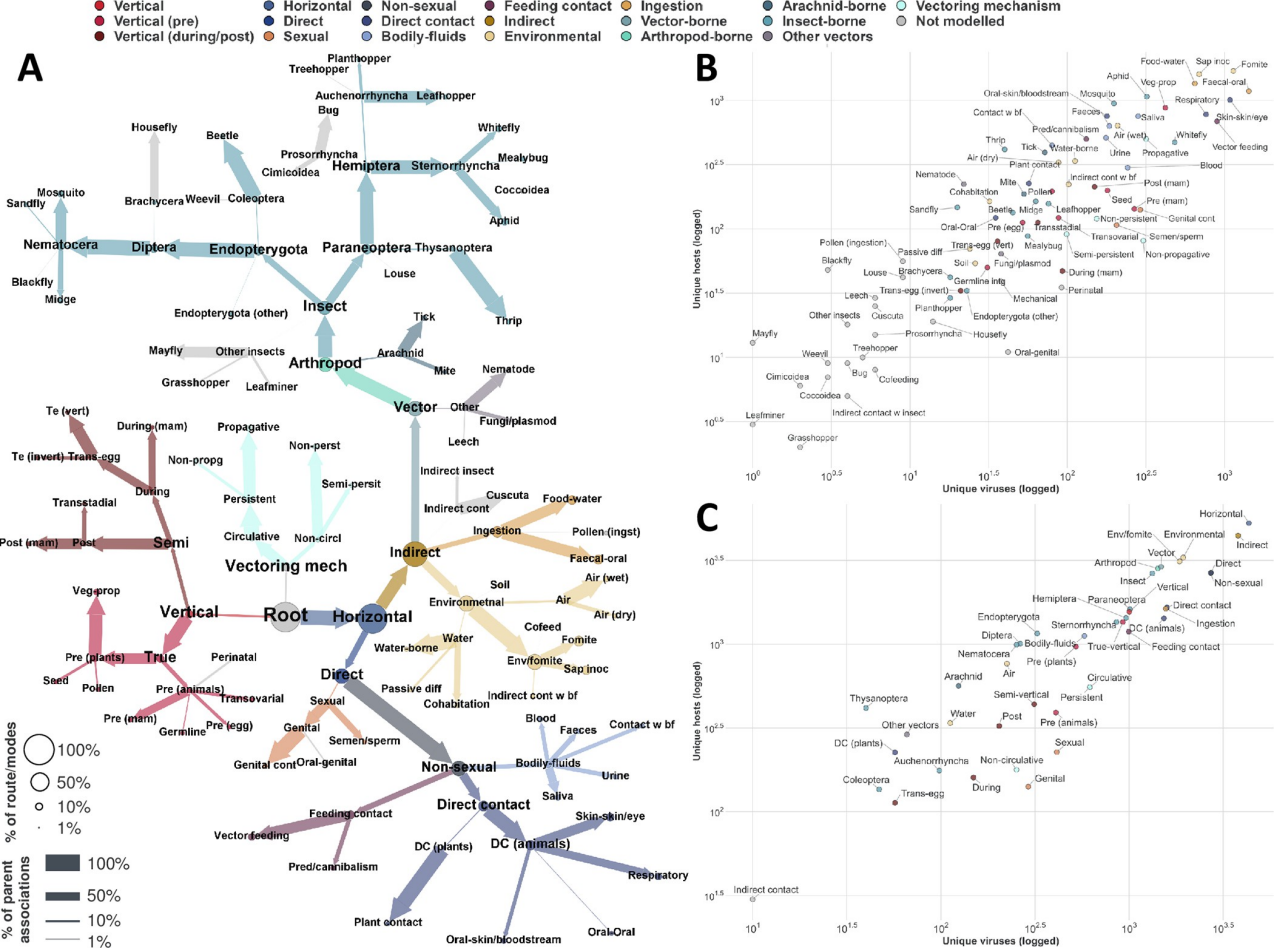
Overview of observed transmission routes/modes. **Panel A–Our proposed hierarchy.** Nodes represent transmission routes/modes identified in this study. Edges link parent modes (nodes with at least one child) with their offspring (e.g. indirect and direct transmission are two modes of horizontal transmission). Nodes and edges are coloured by the mode of transmission; routes not modelled in this study (due to insufficient data, n = 18, modelled routes = 59), and conceptual nodes (root and vectoring mechanism) are coloured in light grey. Node size is proportional to percent of unique virus-host associations (of 24,953 associations) where the virus is transmitted to the host species via the corresponding route/mode. Thickness of edges is proportional to the percent of the parent associations identified to where the virus transmitted to the host species via the child route/mode (e.g. of 10,021 associations transmitted by arthropods [40.16% of included associations], 92.63% are insect-borne, and 9.5% are arachnid-borne). Fig E in S1 Text visualises for each unique route/mode pair (no route/mode in the pair is an ancestor/offspring of the other), the percent of virus-host associations (of total included), whereby the virus is known to be transmitted to the host via both pathways. **Panel B–Transmission routes identified in this study.** Points represent transmission *routes* (Table C in S1 Text) and are coloured by transmission modes. X axis represents the number of observed unique viruses per route. Y axis represents the number of observed unique host species per route. **Panel C–Transmission modes identified in this study.** Given a virus-host association, we considered the virus to be transmitted to the host via a parent *mode* (e.g. Zika virus is insect-borne to humans), if it were transmitted by at least one *route* that is also an offspring/descendant of the parent mode (e.g. zika virus is mosquito-borne to humans), in our hierarchy (Fig 1A).

We captured data on 81 *non-mutually exclusive routes* of 4,446 viruses to 5,317 animal and plant species (a total of 24,953 virus-host associations, Table C in S1 Text, S1 and S2 Datasets), by performing a series of complementary literature searches (see Methods). Where at least one *route* of transmission was identified, we used those data to populate higher levels (*modes*) in our hierarchy. Fig 1 illustrates the distribution of observed transmission routes and modes of virus-host associations across our suggested hierarchy (Fig 1A), as well as between our viruses and hosts (Fig 1B and 1C).

To facilitate the construction of our transmission hierarchy, spanning viruses of humans, animals, and plants, we unified certain transmission pathways into route names that may not be widely used (Table C in S1 Text), these include “Air (dry)” and “Air (wet)”–used to describe transmission via inhalation of virus particles from the environment, but which may be confused with ‘airborne’ transmission, commonly used to describe individual-to-individual transmission via droplets or airborne particles, termed ‘respiratory’ in our study. We elected to separate environmental airborne transmission, from individual-to-individual respiratory transmission as these routes have different epidemiological implications, environmental viruses may persist in the environment for longer period, and do not require direct contact between the individual, for instance hantaviruses are transmissible by inhalation of virus particles from rodent urine (Air (wet)), and many avian viruses are transmitted via inhalation of dust (Air (dry)).

For arthropod-borne viruses, the route is different from vector-to-host compared to host-to-vector, hence, we captured the mechanism of their transmission by the vector to the vertebrate or plant host, as well as from to the arthropod vector to the vertebrate/plant (and between vectors where relevant). For example: Zika virus is mosquito-borne to humans, but mosquitoes become infected with Zika via feeding on humans (arthropod feeding), additionally Zika is transovarially/sexually transmitted in some mosquito species. Additionally, we included the dynamics of vector-transmission, e.g. whether the virus is transmitted mechanically, or if it replicates within the vector, which we termed vectoring mechanism (4 routes, 2 modes). For instance, Tomato Yellow Leaf Curl virus (TYLCV) is whitefly-borne to tomato (and other) plants, and is circulative, non-propagative in its whitefly vector.

### Predictors of transmission routes/modes

**Fig 2:**
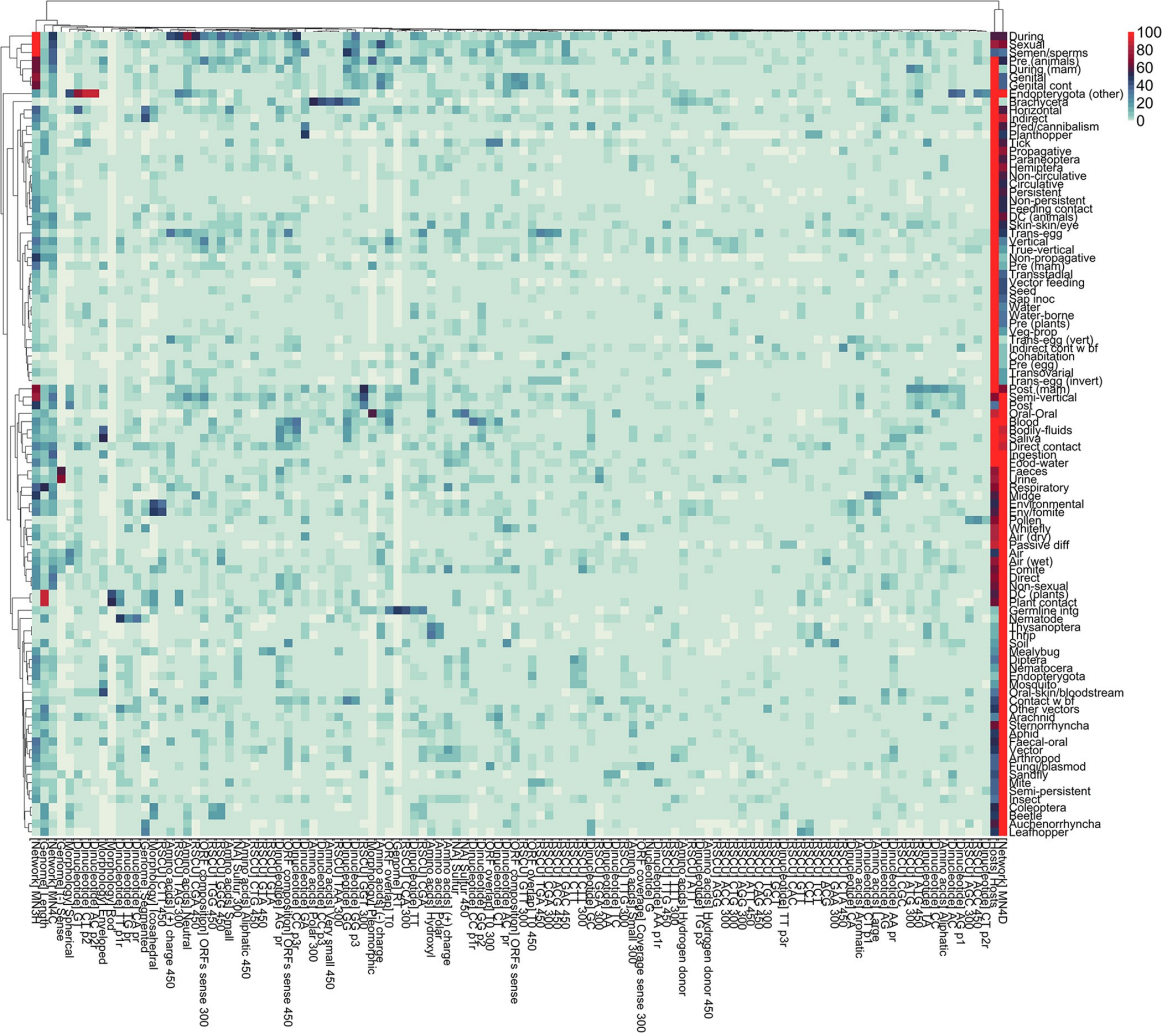
Top 10 predictors of modelled routes/modes. Mean absolute SHAP values were normalised, separately, for each route/mode modelled in this study (scale 1:100, formula = 100*mean SHAP value/max (SHAP value)). Features were ordered by the descending value of the locally normalised SHAP values and the top 10 were selected per each route/mode. The heatmap visualises the contribution (locally normalised SHAP values) the resulting 116 features (Y-axis) made to the predictions of the 98 route/mode modelled in this study (X-axis). We performed hierarchical clustering on both rows and columns, using the R package pheatmap. the resulting dendrogram is displayed (top and left).

Of a total of 446 features, our virus-host integrated neighbourhoods (‘MN4D’ and ‘MN3H’, see Methods and Note 5 in S1 Text) and hosts similarity (‘hosts’) features contributed the most to predictions across all routes/modes (Fig 2). Specifically, ‘MN4D’ was the top predictor of 51.02% of 98 routes/modes with sufficient data for modelling, ‘hosts’ (45.19%), and ‘MN3H’ (3.06%). These three features were in the top ten predictors of 96.94%, 98.98% and 72.45% of our route/modes, respectively (Fig 2 and S3 Data).

Our framework utilised nine genomic structure features (see Table E in S1 Text for details and description of biological relevance), of which the ‘length’ of the virus genome, and whether the virus is ‘segmented’ or not were the most contributing, and were ranked in the top ten predictors of 39.8% and 13.3% of routes/modes, respectively. ‘Length’ made a significant contribution (locally normalised mean absolute SHAP value ≥20) to the predictions of five routes/modes (Fig 2 and S3 Data), including respiratory where it ranked as 3^rd^ predictor. ‘Segmented’ made similar contribution to four routes/modes including Leafhopper-borne transmission where it also ranked 3^rd^ (Fig 2 and S3 Data).

We included 16 virus morphology features (Table E in S1 Text provides details and indicate biological relevance). Five of those features ranked in the top ten predictors of our routes/modes: ‘Enveloped’–categorising if the virus has an envelope or not; and ‘spherical’, ‘pleomorphic’, ‘icosahedral’, and ‘rod’ indicative of the viral structure (capsid). Additionally, ‘Icosahedral’ (17.35%), ‘enveloped’ (14.29%) and ‘spherical’ (10.2%) were in the top ten predictors of >10% of our routes/modes. Our morphological features contributed significantly to the predictions of 14 routes/modes, with ‘enveloped’ making the most significant contribution (Oral-skin/bloodstream transmission, ranked 2^nd^, globally normalised SHAP value = 20.4).

We classified amino acids into 19 overlapping categories expressing various characteristics, and computed biases in those categories, in predicted ORFS, at three different cut-offs, resulting in 57 unique features (Note 3 in S1 Text). Nineteen of our amino acid features were top ten predictors for at least one route/mode, and six were top ten predictors for >10% of our routes/modes: ‘sulphur’ (13.27%), ‘sulphur 450’ (13.27%), ‘(+) charge’ (12.24%), ‘(+) charge 450’ (12.24%), ‘hydroxyl’ (11.22%), and ‘(-) charge’ (10.2%). Our amino acid features made significant contribution to 21 routes/modes, including: ‘(+) charge’ for fomite (ranked 3^rd^) and respiratory transmission (5^th^). The proportion of ‘hydroxyl’ group containing amino acids made the most significant contribution (ranked 2^nd^ for thrip-borne transmission, global SHAP = 17.3).

Of four nucleotide biases, only ‘G’ bias was a top ten predictor (three routes/modes), whereas 32 of 128 dinucleotide biases were top ten predictors for at least one route/mode, with six biases ranked in the top ten predictors for >10% of our models: ‘AG pr’ (AG bias in the reverse complement of the sequence, 14.29%), ‘GT’ (13.27%), ‘GA’ (11.22%), ‘GG’ (11.22%), ‘TT’ (10.2%), and ‘GG p3’ (GG bias in position 3–1 within codon reading frames, 10.2%).

Overall, 116 features were ranked in top ten predictors of each of our routes/modes. S3 Dataset lists the globally and locally normalised contribution (SHAP value) made by the top ten predictors of each route/mode to the prediction of all modelled routes/modes (n = 98).

### Instance-level predictors of transmission routes/modes

**Fig 3:**
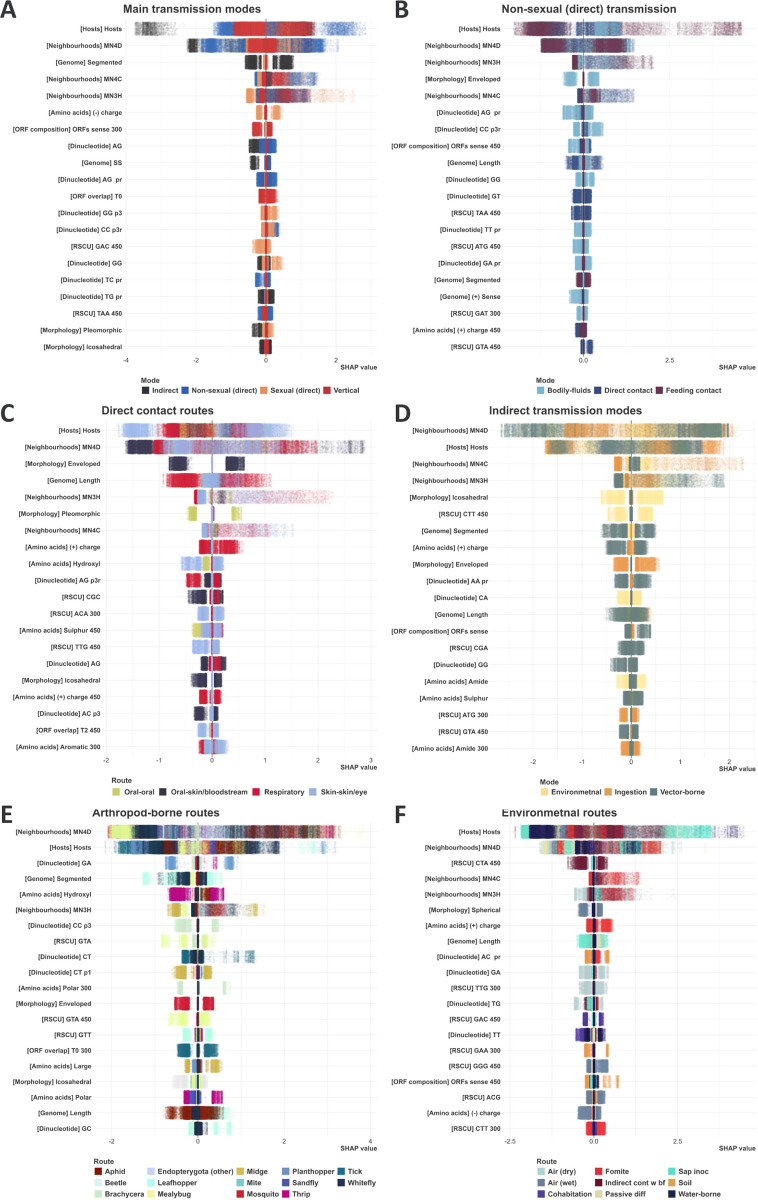
Instance-level feature-contribution to various transmission route/mode prediction. Instance-level SHAP values quantify the contribution each feature made to a particular (virus-host association) prediction. Here, we averaged instance-level SHAP values generated by all constituent models of each of our top-10 ensembles (50 per route/mode). In each sub-plot, features were ordered by the spread of their variance (max(variance)-min(variance) across all routes/modes included in each sub-plot), and the top 20 features (from most to least spread) were selected. Points represent virus-host associations (instances) and are coloured by the underlying route/mode. The Y axes represent the selected features (category of each feature between brackets). The X axes represent SHAP values. Positive SHAP values indicate that the feature has contributed towards a positive prediction for the virus-host association (the virus species/strain is transmitted to the host species via the given route/mode). Negative SHAP values indicate that the feature has contributed towards a negative prediction (the virus is not transmitted to the host via route/mode). Larger magnitudes indicate that the feature has had a stronger influence on the prediction for the particular instance.

Fig 3 visualises the instance-level contributions of top twenty features, by spread of variance in each sub-plot/category, for six categories: main transmission modes (3.A), direct (non-sexual) transmission modes (3.B), direct contact routes (3.C), indirect transmission modes (3.D), arthropod-borne routes (3.E), and environmental transmission routes (3.F). Overall, our virus-host integrated neighbourhoods (MN4D) and hosts similarity features had the most spread of variance across the six categories, whereas the spread of our viral features varied per category.

‘Segmented’ was the viral feature with most spread in variance of contribution to our main modes of transmission (Fig 3A and S3 Data); ranked 3^rd^ predictor of ‘indirect’ mode of transmission, but only 18^th^, 42^nd^, and 87^th^ predictor of ‘sexual’, ‘non-sexual’, and ‘vertical’ transmission modes, respectively. ‘Enveloped’ had the most spread in variance for both direct transmission modes (Fig 3B) and direct contact routes (Fig 3C), ranking 3^rd^ for ‘bodily-fluids’ transmission mode and 2^nd^ for ‘oral-skin/bloodstream’ transmission route, but having very little to virtually no impact on the remaining modes and routes.

S3 Dataset lists full mean SHAP value, variance, and spread for all features included in Fig 3. Figs H-L in S1 Text visualise the remainder routes/modes not included in Fig 3.

### Prediction of potential routes/modes

**Fig 4:**
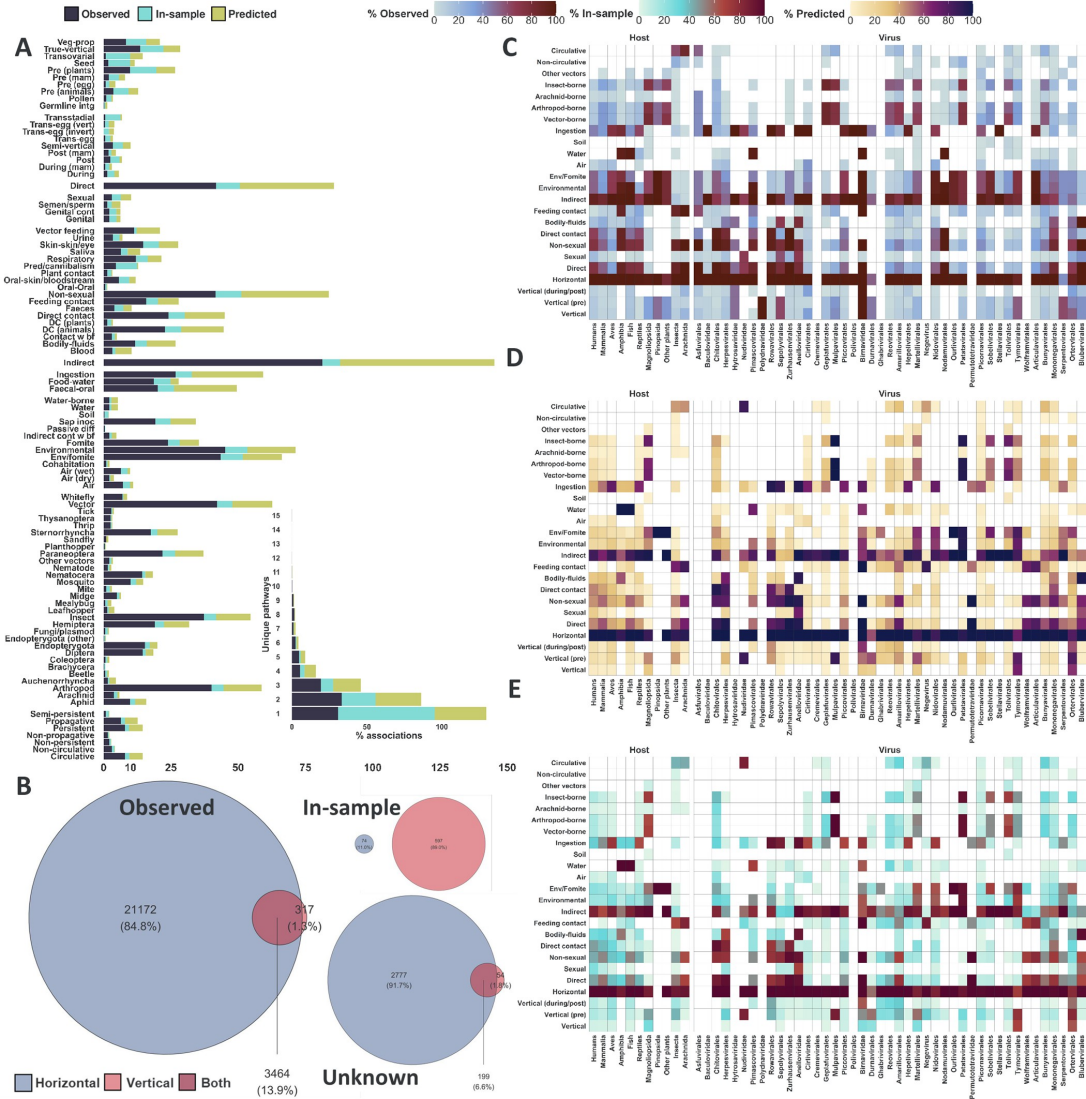
Predicted transmission routes and modes. Panel A–Proportion of predicted unknowns (yellow, n = 3,108, no known transmission route, mean probability cut-off>0.5), in-sample predictions (cyan, n = 6,701, hitherto unobserved routes predicted with mean probability cut-off>0.5 for associations with at least one observed route) and observed virus-host instances (dark blue, n = 24,953, at least one observed transmission route). Vertical and horizontal modes were removed from the bar plot for better visualisation (represented in panel B as Venn diagrams). The inset (bottom) represent the percent of unique pathways for unknowns and in-sample predictions, as well as observed for each virus-host association. Figs E, F, and G in S1 Text visualise the percent of virus-host associations, whereby the virus is observed, predicted within sample, and predicted for unknowns, to be transmitted to the host via each pair of unique pathways, respectively. **Panel B–Horizontal and vertical transmission.** Venn diagrams represent horizontal transmission (blue) and vector transmission modes (red) for observed, in-sample predictions (with at least one previously observed route, but that is not observed to be transmitted via the corresponding mode), and unknown (out of sample) predictions (instances without observed routes), respectively. **Panel C–Proportion of host-virus instances transmitted by each main route/mode per each host group or virus order.** Rows represent main transmission modes/routes. Columns represent main host groups, and virus orders. Proportions are calculated by the number of instances known to be transmitted via given route/mode per each category (e.g. humans), divided by the total number of instances in the category. Some routes/modes were grouped together for better visualisation (e.g. Vertical (pre), insect-borne). **Panel D–Proportion of host-virus instances, without previously observed transmission route, predicted to be transmitted by each main route/mode per each host group or virus order.** Rows represent main transmission modes/routes. Columns represent main host groups, and virus orders. Proportions are calculated by the number of instances predicted to be transmitted via given route/mode per each category, divided by the total number of instances in the category. **Panel E–Proportion of host-virus instances, with at least one previously observed transmission route, predicted to be transmitted by each main route/mode per each host group or virus order.** Rows represent main transmission modes/routes. Columns represent main host groups, and virus orders. Proportions are calculated by the number of instances predicted to be transmitted via given route/mode per each category, divided by the total number of instances in the category.

Our framework predicted (mean top-10 ensemble probability > 0.5) at least one route/mode for 3,108 out of 3,708 (83.82%) virus-host instances without known routes in our dataset (2,004 viruses (8.1.63%) to 1,300 host species (87.84%)) (Fig 4). Of those instances, 2,969 were predicted to be transmitted horizontally (80.07% vs 98.73% of observed associations), and 249 were predicted to be transmitted vertically (6.715% vs 15.15%). Indirect (57.50% vs 81.25%), direct (34.98% vs 41.77%), non-sexual (32.42% vs 41.65%), and ingestion (26.54% vs 26.76%) *modes* were the most predicted after horizontal transmission. The faecal-oral route was the most predicted route of transmission for unknown associations (23.33% vs 20.07%), followed by sap inoculation (9.47% vs 19.36%—a plant-only route).

Additionally, our framework made in-sample predictions (routes/modes predicted with mean top-10 ensemble probability>0.5 for virus-host associations with at least one observed transmission route in our dataset) for 6,701 virus-host associations (26.85% of total associations). The top additional routes/modes predicted in-sample were as follows: true vertical transmission in plants—pre-plants (649 additional associations, 9.685% of total in-sample predictions vs 9.86% of observed and 6.96% of predicted for unknowns) and non-sexual (direct) transmission (639, 9.535% vs 41.65% and 32.42%). Fig 4 visualises both in-sample as well as out-of-sample (unknowns) predicted routes/modes.

Given a virus-host association, we constructed a representative set of *unique transmission pathways* by traversing our hierarchy (Fig 1A) from routes to root, and including all transmission *routes* of the given virus to the focal host, as well as any transmission *modes* that are not ancestors of any already included routes/modes. We predicted that 1,068 instances have a single unique transmission pathway (29.33% of unknown instances vs 30.89% of associations with at least one observed route and 64.525% for our in-sample predictions); 953 (26.17% vs 33.25% and 22.53%) to have two unique pathways; and 1,020 (28.01% vs 35.855% and 12.94%) to have three or more unique pathways.

### Prediction dependencies

**Fig 5:**
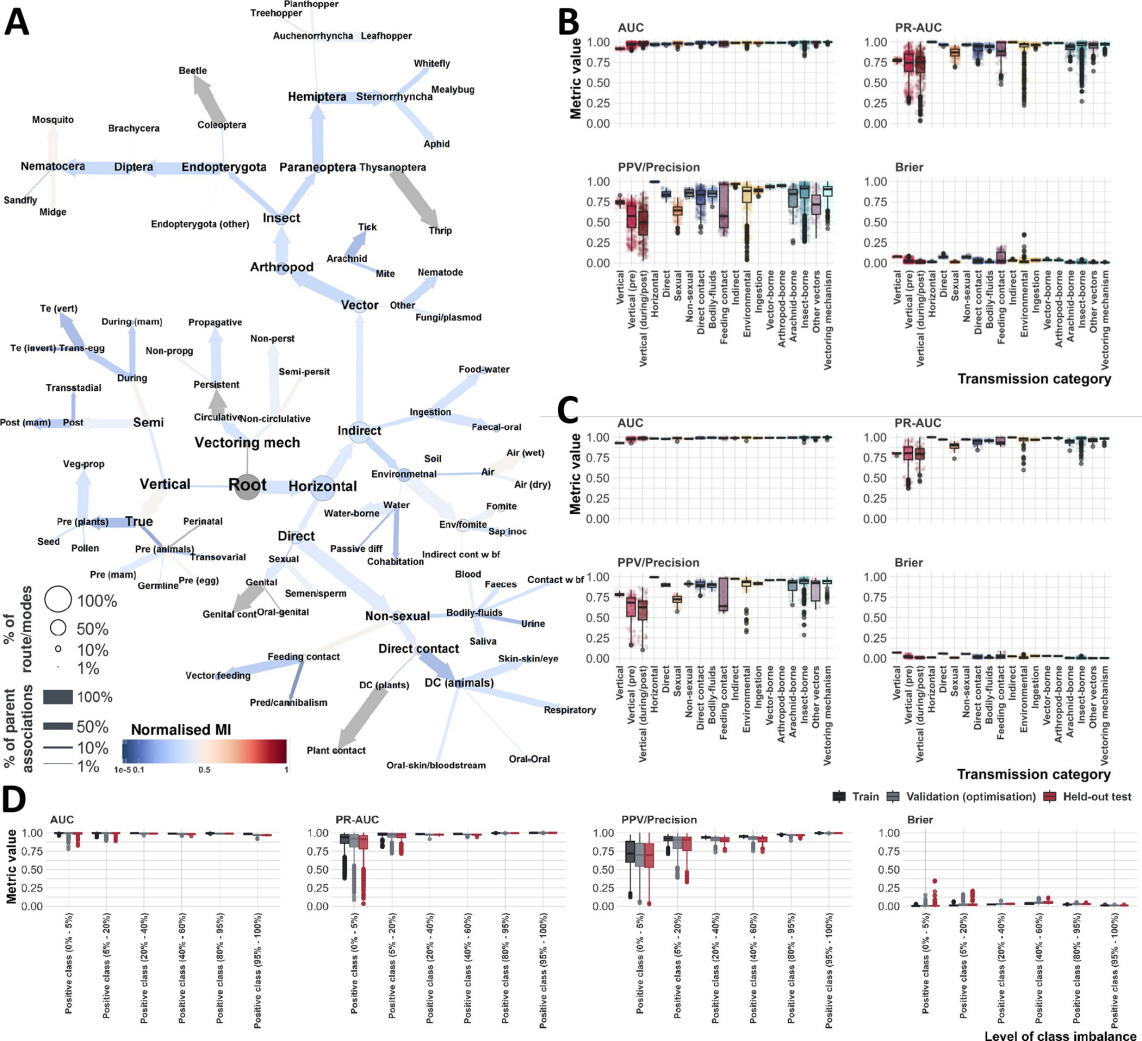
Prediction dependencies and performance assessment. **Panel A–Prediction dependencies**. Nodes represent transmission routes/modes modelled in this study. Edges indicate our hierarchy (Fig 1). Nodes are coloured by Normalised Mutual Information (MI) estimates between the mean probabilities (derived from our top-10 ensembles) of instances predicted by the route/mode represented by the node (mean probability > 0.5), and corresponding instance-wise probabilities of their siblings (nodes with the same parent node). Grey nodes indicate structural nodes (not transmission related), and only children (routes without modelled siblings). Nodes sizes and thickness of edges are same as Fig 1. MI quantifies how much knowing the value of one variable can tell us about the value of the other variable. If the resulting estimate is high, it indicates a strong relationship or dependency between the variables, meaning that knowing one variable provides useful information about the other. To assess the statistical significance of the MI estimates, we compared each estimate to a null distribution using bootstrapping (n = 2,000). Fig O in S1 Text visualises the resulting p-values. Figs P-Q in S1 Text visualise dependencies between probabilities for routes/modes and knowledge of their siblings, predictions of their siblings, and knowledge of routes/modes and resulting probabilities of their siblings, respectively. **Panel B–Performance assessment of constituent class-balancing ensembles on held-out test sets.** Points represent the class-balancing ensemble mean values for each performance metric (50 points per route/mode, 98 routes/modes). Figs S and U in S1 Text illustrate results performance assessment using ten metrics, for all class-balancing ensembles, and their constituent models, respectively. **Panel C–Performance assessment of our top-10 selected ensembles on held-out test sets.** Points represent the class-balancing ensemble mean values for each performance metric (10 points per route/mode). Figs T and V in S1 Text illustrate performance assessment, using ten metrics, for all top-10 ensembles, and their constituent models, respectively. In panels B and C, Boxplots represent the interquartile range (IQR), of the data distribution per each category of transmission route/mode. Horizontal lines within the box represent the median of the data distribution. Whiskers extend from the edges of the box to the minimum and maximum values within 1.5 times the IQR from the nearest quartile, individual data points that fall outside the range covered by the whiskers are plotted as outliers. For Brier score values closer to 0 indicate better performance, and those closer to 1 indicate worse performance. **Panel D–Performance assessment of class-balancing ensembles on training, validation, and held-out test sets, per level of class imbalance.** Boxplots represent the interquartile range (IQR), of the data distribution per training (dark grey), validation (light grey), and test (red) sets. Horizontal lines within the box represent the median of the data distribution. Whiskers extend from the edges of the box to the minimum and maximum values within 1.5 times the IQR from the nearest quartile, individual data points that fall outside the range covered by the whiskers are plotted as outliers. Table H in S1 Text provides full definitions of included performance metrics. Fig X in S1 Text visualises training, validation, and test set performance per category of class imbalance using ten metrics.

We utilised Mutual Information (MI) to quantify the relationship between predictions (top-10 ensemble mean probability > 0.5) for a given route/mode and those of its sibling route(s)/modes(s)—children of the same parent node in our hierarchy (Fig 1). Fig 5A visualises the resulting normalised MI estimates. Our normalised MI ranged between 0.00002 (predation/cannibalism) and 0.027 (Brachycera-borne), suggesting a very weak to weak correlation (very limited to limited relationship or dependency) between the predictions of the focal route and those of its siblings. The routes with highest normalised MI were: Brachycera-borne (0.027) and air (wet) (0.025).

### Performance assessment

We employed a random cross-validation strategy, over 50 iterations, for each route/mode (n = 98). In each iteration, the training set was balanced using five different resampling approaches, resulting in five distinct balanced sets. We trained a LightGBM model for each set, tuning it using the same validation set (10% of available data, different per iteration and route/mode combination, see methods). We then averaged the resulting probabilities to generate a bagging ensemble (termed class-balancing ensemble) per each iteration and route/mode combination. We evaluated the performance of each ensemble against the corresponding held-out test set (15% of available data, different per iteration and route/mode combination; see Methods).

Overall, our framework achieved an average ROC-AUC = 0.988±0.017, and an average F1-score = 0.806±0.169. High ROC-AUC values indicate strong ability in distinguishing between positive and negative instances. Conversely, high F1-scores highlight the effectiveness in identifying positive instances while balancing precision and recall, thereby minimising potentially false positives and false negatives. Table 1 lists the average performance metrics across training, validation, and held-out test sets, obtained from all class-balancing ensembles (n = 4,900).

**Table 1:** Average performance metrics across training, validation, and held-out test sets for all class-balancing ensembles and test set performance for top-10 ensembles, for all routes/modes. The average performance is calculated as the mean over 50 iterations for the training, validation, and test sets, and over 10 iterations for the top-10 ensembles. Except for ROC-AUC and PR-AUC, all other metrics were computed at >0.5 probability threshold. The top-10 ensembles were selected by ranking each route/mode class-balancing ensembles (n = 50) based on the average of four metrics—AUC, PR-AUC, PPV/Precision, and adjusted Brier score (1—actual score)—computed on the test sets, and then selecting the best 20% ranked ensembles. Brier scores range from 0 (best performance) to 1 (worst performance), while MCC values range from +1 (best performance) to -1 (worst performance). ± values indicate standard deviation from the mean. Values in square brackets indicate the worst and best performing ensembles, respectively. S4 Dataset provides the average performance metrics (and their standard deviations) across the training, validation, and held-out test sets, as well as the percentage of positive class instances for each route/mode.

Metric	Training sets	Validation sets	Test sets	Top-10 (test sets)
**ROC-AUC**	0.994±0.009 [vertical = 0.944, brachycera-borne = 0.999]	0.989±0.017 [vertical = 0.921, cohabitation = 0.999]	0.988±0.017 [vertical = 0.917, cohabitation = 0.999]	0.991±0.012 [Vertical = 0.931, Cohabitation = 0.999]
**PR-AUC**	0.928±0.101 [transovarial = 0.332, horizontal = 0.999]	0.901±0.14 [transovarial = 0.358, horizontal = 0.999]	0.891±0.147 [trans-egg (invert) = 0.332, horizontal = 0.999]	0.922±0.109 [transovarial = 0.446, horizontal = 0.999]
**F1-score**	0.847±0.143 [transovarial = 0.350, horizontal = 0.994]	0.814±0.163 [Trans-egg (invert) = 0.282, horizontal = 0.994]	0.806±0.169 [Trans-egg (invert) = 0.237, horizontal = 0.994]	0.855±0.143 [Trans-egg (invert) = 0.293, horizontal = 0.995]
**NPV**	0.988±0.048 [horizontal = 0.541, passive diffusion = 0.999]	0.986±0.05 [horizontal = 0.524, cohabitation = 0.999]	0.987±0.048 [horizontal = 0.546, mealybug-borne = 0.999]	0.987±0.042 [horizontal = 0.601, passive diffusion = 0.999]
**PPV/ Precision**	0.797±0.184 [transovarial = 0.222, horizontal = 0.998]	0.764±0.2 [trans-egg (invert) = 0.178, horizontal = 0.995]	0.755±0.206 [trans-egg (invert) = 0.149, horizontal = 0.995]	0.829±0.179 [trans-egg (invert) = 0.180, horizontal = 0.996]
**Recall/ Sensitivity**	0.929±0.065 [vertical = 0.670, vector feeding = 0.996]	0.900±0.105 [seed = 0.605, vector feeding = 0.996]	0.896±0.105 [seed = 0.605, vector feeding = 0.995]	0.905±0.09 [seed = 0.615, vector feeding = 0.995]
**Specificity**	0.987±0.022 [horizontal = 0.814, sandfly-borne = 0.999]	0.979±0.044 [horizontal = 0.651, sandfly -borne = 0.999]	0.976±0.052 [horizontal = 0.656, sandfly -borne = 0.999]	0.984±0.037 [horizontal = 0.672, sandfly -borne = 0.999]
**MCC**	0.842±0.128 [transovarial = 0.423, vector feeding = 0.983]	0.806±0.15 [trans-egg (invert) = 0.356, vector feeding = 0.980]	0.797±0.156 [trans-egg (invert) = 0.305, vector feeding = 0.982]	0.847±0.133 [trans-egg (invert) = 0.384, vector feeding = 0.990]
**Brier**	0.013±0.013 [vertical = 0.061, passive diffusion = 0.001]	0.017±0.018 [feeding contact = 0.074, sandfly-borne = 0.002]	0.018±0.024 [feeding contact = 0.144, sandfly-borne = 0.002]	0.014±0.016 [vertical = 0.070, passive diffusion = 0.001]
**TSS**	0.915±0.07 [vertical = 0.669, vector feeding = 0.993]	0.879±0.111 [seed = 0.578, vector feeding = 0.992]	0.872±0.111 [seed = 0.578, vector feeding = 0.992]	0.889±0.096 [seed = 0.593, vector feeding = 0.994]

To identify the most robust models, we ranked each route/mode class-balancing ensembles (n = 50, see Methods), based on the average of four metrics: ROC-AUC, PR-AUC, Precision, and 1-Brier score, measured using the held-out test sets. We selected the top 20% as the top-10 performing ensembles to generate final predictions. Our top-10 ensembles achieved an average ROC-AUC = 0.991±0.012, and an average F1-score = 0.855±0.143. Table 1 lists the average performance of our top-10 ensembles against ten metrics. Fig 5B and 5C visualise the performance of all class-balancing ensembles, and our top-10 ensembles, respectively. Fig W is S1 Text provides post-hoc assessment of in-sample predictions of our top-10 selected ensembles.

As the proportion of virus-host associations where the virus is transmitted to the host via a given route/mode varied considerably between different routes (S4 Dataset, Methods, and Fig C in S1 Text), we evaluated the performance of our class-balancing ensembles, and their constituent models, across multiple levels of class imbalance. We categorised those levels into six distinct ranges, reflecting the distribution of classes for our routes/modes (almost all negative [0%-5%], mostly negative [5%-20%], more negative than positive [20%-40%], almost balanced [40%-60%], mostly positive [80%-95%], and almost all positive [95%-100%]). Fig 5D illustrates the performance assessment of our class-balancing ensembles on training, validation, and held-out test sets, per level of class imbalance, using our selection metrics. Fig X in S1 Text visualises the results of the same performance assessment for ten metrics. Table 2 summarises performance assessment, across ten metrics, categorised by levels of class imbalance, for training, validation, and test sets. Figs Y and Z in S1 Text illustrate the ranking of held-out test set performance of our bagging ensembles and their constituent class-balancing models, as well as pairwise comparison of performance, respectively. Fig AA in S1 Text illustrates the difference in performance between validation and test sets, per level of class imbalance, for all class-balancing ensembles. Fig AB in S1 Text visualises variance in performance, over 50 iterations, for all class-balancing ensembles. Table I in S1 Text lists average absolute percent difference in performance between validation and test sets for our ensembles and their constituent models. Table J in S1 Text lists the average variance in performance of our ensembles, and their constituent models, over 50 iterations.

**Table 2:** Average performance metrics, per level of class imbalance, across training, validation, and held-out test sets for all class-balancing ensembles and all routes/modes. The average performance is calculated as the mean over 50 iterations for the training, validation, and test sets, and over 10 iterations for the top-10 ensembles. Except for ROC-AUC and PR-AUC, all other metrics were computed at >0.5 probability threshold. Brier scores range from 0 (best performance) to 1 (worst performance), while MCC values range from +1 (best performance) to -1 (worst performance). ± values indicate standard deviation from the mean. Values in square brackets indicate the worst and best performing ensembles, respectively. Fig AA in S1 Text visualises difference in performance between held-out test sets and validation sets, per level of class imbalance, for all class-balancing ensembles and their constituent models. Fig AB in S1 Text illustrates variance in performance on the test-sets across the 50 iterations, per level of class imbalance, for all class-balancing ensembles and their constituent models.

Split	Class imbalance	ROC-AUC	PR-AUC	F1-score	NPV	PPV/ Precision	Recall/ Sensitivity	Specificity	MCC	Brier	TSS
**Training**	**[0% - 5%]**	0.995±0.006	0.897±0.119	0.787±0.155	0.999±0.002	0.708±0.192	0.918±0.067	0.994±0.006	0.796±0.139	0.006±0.005	0.913±0.071
	**[5% - 20%]**	0.992±0.014	0.963±0.049	0.92±0.065	0.991±0.012	0.905±0.065	0.938±0.073	0.987±0.009	0.91±0.073	0.017±0.014	0.926±0.08
	**[20% - 40%]**	0.994±0.003	0.983±0.007	0.942±0.015	0.982±0.008	0.937±0.015	0.947±0.021	0.979±0.004	0.922±0.018	0.025±0.007	0.926±0.023
	**[40% - 60%]**	0.989±0.004	0.985±0.006	0.947±0.015	0.96±0.013	0.95±0.013	0.945±0.019	0.963±0.01	0.909±0.026	0.039±0.01	0.908±0.027
	**[80% - 95%]**	0.992±0.001	0.998±0.0003	0.985±0.001	0.96±0.003	0.979±0.003	0.991±0.001	0.907±0.011	0.918±0.008	0.022±0.002	0.898±0.012
	**[95% - 100%]**	0.985±0.002	0.999±0.0001	0.994±0.001	0.541±0.065	0.998±0.001	0.991±0.002	0.814±0.021	0.657±0.042	0.012±0.002	0.804±0.021
**Validation**	**[0% - 5%]**	0.99±0.016	0.859±0.165	0.749±0.176	0.998±0.003	0.677±0.21	0.877±0.118	0.993±0.01	0.758±0.163	0.008±0.008	0.87±0.121
	**[5% - 20%]**	0.988±0.02	0.948±0.064	0.888±0.091	0.989±0.014	0.862±0.108	0.923±0.087	0.978±0.028	0.874±0.101	0.023±0.023	0.901±0.099
	**[20% - 40%]**	0.991±0.004	0.974±0.011	0.923±0.024	0.978±0.012	0.912±0.036	0.936±0.033	0.97±0.013	0.897±0.03	0.032±0.009	0.906±0.034
	**[40% - 60%]**	0.983±0.007	0.977±0.01	0.93±0.024	0.957±0.013	0.918±0.041	0.943±0.017	0.937±0.036	0.878±0.043	0.05±0.016	0.88±0.041
	**[80% - 95%]**	0.988±0.003	0.997±0.001	0.978±0.007	0.949±0.016	0.967±0.013	0.989±0.004	0.854±0.064	0.878±0.041	0.029±0.005	0.843±0.063
	**[95% - 100%]**	0.967±0.017	0.999±0.0004	0.994±0.002	0.524±0.09	0.995±0.001	0.992±0.003	0.651±0.086	0.575±0.069	0.013±0.002	0.643±0.085
**Test**	**[0% - 5%]**	0.988±0.016	0.844±0.173	0.741±0.182	0.998±0.003	0.67±0.216	0.869±0.116	0.992±0.022	0.75±0.169	0.009±0.014	0.861±0.121
	**[5% - 20%]**	0.987±0.022	0.944±0.065	0.878±0.105	0.989±0.014	0.847±0.132	0.923±0.085	0.972±0.05	0.864±0.116	0.027±0.032	0.895±0.102
	**[20% - 40%]**	0.99±0.004	0.973±0.01	0.921±0.02	0.979±0.01	0.903±0.036	0.941±0.029	0.966±0.013	0.894±0.025	0.034±0.008	0.907±0.027
	**[40% - 60%]**	0.982±0.008	0.976±0.01	0.925±0.028	0.959±0.011	0.906±0.049	0.947±0.014	0.925±0.043	0.868±0.049	0.053±0.019	0.872±0.046
	**[80% - 95%]**	0.988±0.003	0.997±0.001	0.976±0.006	0.944±0.018	0.965±0.013	0.988±0.004	0.843±0.064	0.869±0.038	0.031±0.006	0.831±0.062
	**[95% - 100%]**	0.968±0.012	0.999±0.0003	0.994±0.001	0.546±0.091	0.995±0.001	0.992±0.003	0.656±0.066	0.59±0.055	0.013±0.002	0.648±0.065

Additionally, we retrained two separate suites of models using plant-only focusing on routes/modes that affect plants and animal-only data for routes/modes which affect animals. We found no significant difference in performance between models trained using both animal and plant data, and those using animal-only, or plant-only data. Fig AC in S1 Text visualises performance of all class-balancing ensembles trained with animal and plant data, ensembles trained with animal-only data, and ensembles trained with plant-only data. Tables K-M in S1 Text compare the average performance of each subset of models (trained with both animal and plant data, animal-only, or plant-only data).

## Discussion

In this study, we constructed a computational framework that explored the landscape of viral transmission in the animal and plant kingdoms, with the aim to firstly uncover the specific viral features and evolutionary signatures predictive of the transmission routes; secondly to assess the applicability of predictive approaches as means to triage the potential transmission routes of emerging viruses; and finally to quantify possible gaps in our knowledge of transmission pathways of existing viruses to their known hosts.

This was achieved by training lightGBM ensembles on a comprehensive dataset of transmission routes and modes of 4,446 viruses to 5,317 animal and plant species (Fig 1). Broadly, 112 of our 442 viral features were important predictors for at least one of 98 routes and modes of transmission analysed (Fig 2). Furthermore, analysing the differences in contribution our viral features made to individual predictions of hierarchically close routes and modes (Fig 3), enabled us to establish the different roles the same, or similar, viral features and evolutionary signatures play in influencing viral transmission dynamics. We further quantified the ability of our ensembles to discriminate between closely related routes/modes by examining dependencies of their predictions (Fig 5A), and found that overall, our ensembles exhibited very limited to limited dependency between related routes/modes.

Overall, our approach utilising independent class-balancing ensembles per each route/mode, performed well across varying levels of class imbalance, averaging F1-score of 0.741 and 0.878 when the present of positive class is almost all negative [0%-5%] and mostly negative [5%-20%] respectively (Tables 1 and 2 and S4 Dataset). The bagging of models trained with different class-balancing sampling approaching improved overall predictive performance, compared with using any single approach, and also reduced overfitting by averaging the predictions from multiple models. Additionally, our ensembles achieved high level of performance for routes commonly associated with high consequence human, animal, and plant viruses (e.g. vector-borne, respiratory viruses).

We applied our ensembles to predict the transmission routes of 2,004 virus species or strains for which there are no known transmission routes to 1,300 host species. Our models predicted at least one route/mode for ~84% of those instances (Fig 4). Furthermore, we identified an additional 19,396 transmission routes/modes potentially un-observed in virus-host associations with at least one route/mode observed (Fig 4). These predictions were made across a total of 4,076 animal and plant viruses.

This study, therefore, showcases the potential to provide early insights into the epidemiology of a newly emerging virus, and hence can be used to facilitate rapid response and significantly triage the time-consuming investigations to confirm the routes.

### Application of multiple perspective features in predicting transmission routes

We generated predictive features from three complementary perspectives: viruses, hosts, and our virus-host integrated neighbourhoods which depict the topology of the virus-host network in the phylogenetic neighbourhood [28] of a virus.

Our integrated neighbourhoods and host similarity features were highly predictive of all transmission routes/modes (Fig 2). However, all of our viral features were also predictive of at least one route/mode. This highlights the advantage of our multi-perspective approach to investigating mechanisms of virus transmission, and further emphasises the applicability of multi-perspective approaches, in line with the significant promise they have previously shown in predicting virus-host associations [1,2,29,30].

Other existing approaches which aim to predict viral phenotypes solely from one perspective–such as the viral sequence–will miss key features from the host and network perspectives which would enhance their predictive performance. For example, our integrated neighbourhoods and host features were the most informative predictors across all routes/modes, providing the large-scale structure of the viral transmission landscape. Our host similarity was a top-10 predictor of 97/98 routes.

Our 442 viral features (often referred to as viral evolutionary signatures [28]) further enhanced accuracy and explainability at higher resolutions of the individual association and route/mode levels, and therefore improving distinction between similar sister routes/modes. For instance, envelope status was an important predictor of mosquito-borne transmission (ranked 3^rd^) but has no effect on predicting sister routes midge- and sandfly-borne transmission. Conversely, ‘CT p1’ bias (refer to Note 3 ins S1 Text and Table E in S1 Text for full definition) was predictive of midge-borne transmission but has no effect on predicting mosquito-borne transmission, and negligible effect on predicting sandfly-borne transmission. This is mirrored throughout our hierarchy, including routes that are often interlinked: For example, and in line with previous studies [27], envelop status was a top predictor of faecal-oral transmission, but has no effect on predicting transmission via ingestion of food/water (Fig L in S1 Text).

### Strength of predictions in potentially high-consequence transmission routes

Routes of transmission affect virus ecology and epidemiology [10]; determining the virus spread within and between host species and populations, and, for some routes, the geographical range of outbreaks [33,34]. Respiratory (in humans) and vector-borne (in animal and plants) transmission frequently results in high-consequence outbreaks [12,16,35], and our framework is particularly effective at identifying viruses with these two mechanisms. For example, for respiratory viruses, our top-10 ensembles approach achieved mean ROC-AUC = 0.990, and mean F1-Score = 0.864; while our arthropod-borne classifiers averaged ROC-AUC = 0.997, and F1-score = 0.921 (S4 Dataset). Furthermore, at the level of individual vector-borne routes, our top-10 ensembles exhibited high predictive performance for all important routes of both plant and animal viruses (S4 Dataset). This strength is likely driven by the large amount of data (e.g. 40% of our virus-host associations had at least one arthropod-borne route of transmission), and the large number of human-virus data assigned to respiratory transmission (33% of virus-human associations). Given the importance of these two classes from previous high-consequence outbreaks, the high density of data serves to improve our pipeline’s predictive performance where it is needed most.

### Re-predicting secondary routes of high consequence viruses

One key application of this study is to uncover important secondary transmission routes for high consequence viruses, especially with a goal of being able to predict these routes early in an outbreak. To demonstrate this utility, we opted to systematically select exemplar viruses based on heath/economic importance and recent outbreaks, across the breadth of our hosts to demonstrate the potential application. We used our held-out test sets to assess the ability of our framework to re-predict these important routes (see Methods).

During the most recent large outbreak of Zaire Ebolavirus, 2014–2016, in mid-2015 the potential for Ebola to be sexually transmitted was still being debated, but with insufficient evidence either way [36]. Finally, in December that year, molecular evidence was discovered [37]. Here, our framework re-predicts the sexual transmission of Zaire Ebolavirus at 0.52 probability (0.50 via semen, S4 Dataset). Similarly, for Zika virus, it was not until half way through the 2015–2017 outbreak in the Americas that sufficient evidence for confirmation of sexual transmission was obtained [8]. Again, our framework re-predicts sexual transmission of Zika virus at 0.53 (0.53 via semen). These re-predictions from held-out instances demonstrate that even for epidemiologically minor secondary routes of transmission, sufficient signal is still detected to highlight potential additional routes for further study and/or mitigation advice for ongoing outbreaks.

The zoonotic Nipah Henipavirus was traditionally considered as circulating between animals by being transmitted directly from infected animal to animal [38]. However, in Bangladesh, the natural reservoir, *Pteropus* bats, have only once been implicated in direct transmission of Nipah to pigs, despite high seroprevalence in suids. It was later shown that contaminated food (date palm sap) is the primary route of transmission, with the trees having become contaminated from bats prior to harvest [39]. Our framework strongly re-predicts this food-borne transmission at 0.70 (to *Sus scrofa*). Further, this insight later informed the discovery of food-borne transmission to humans. Our framework did, however, fail to re-predict airborne transmission (via wet or dry particles in the air) of Porcine epidemic diarrhea virus (to *Sus scrofa*), with only 0.13 for air (wet). This is both a well-studied route and host with a high amount of data for each, and is a common transmission route for this virus [40]. Therefore, our methodology does fail in some instances.

In plants, maize chlorotic mottle virus is vectored by adult and larval chrysomelid beetles. It was first reported in Peru in 1973, later spread across South and North America, and continues to expand its range beyond the New World [17]. More recently, vertical transmission in up to 1% of seeds has been discovered in outbreaks in Hawaii [41] and Tanzania [42]. Our framework re-predicts seed transmission (to *Zea mays*) at 0.79 probability. Additionally, cassava brown streak virus, which is endemic to Eastern Africa is considered the single biggest viral threat to food security [43]. Primarily vectored by the whitefly *Bemisia tabaci*, it was first suspected that it could also be vertically transmitted through stem cuttings when the cassava crop was reintroduced to Tanzania [44]. Our pipeline successfully re-predicts transmission by vegetative propagation at 0.76 (to *Manihot esculenta*).

Of all six high-consequence viruses/routes selected, our framework correctly re-predicted five. We presume our failure to re-predict Porcine epidemic diarrhea virus is likely to be due to the potential of multiple different evolutionary strategies leading to the same transmission route, and consequently multiple different sets features which would appear as noise in our training data. In future, as more data become available from different strategies/feature sets, our pipeline will become more able to distinguish these–but as of now we are limited by the data available. Overall, however, of the high-consequence viruses assessed, and including both common and epidemiological minor secondary routes, our pipeline is remarkably capable of re-predicting known routes from hold-out sets and taken together with our performance metrics, appears to be able to identify unknown secondary routes in novel viruses with a high degree of accuracy.

### Framework deployment to associations without any known routes

We predicted transmission mechanisms for viruses with no observed routes to their known hosts. Proportionally, direct transmission routes were overall more likely to be predicted than indirect (Fig 4). However, ingestion and faecal-oral were the most likely unobserved individual routes to be predicted. We also noted a significant underestimation in the number of plant viruses potentially transmitted by insect.

The proportion of predicted to observed transmission routes was greatest for arthropod-borne routes and modes, with the Hemiptera-borne routes having the most predicted associations; the Hemiptera are a superorder of insects which contains the majority of the plant vectors [31]. Given the relative difficulty, time, and expense of demonstrating arthropod-borne transmission, which requires vector competence studies [32], it is not surprising that many such routes remain undetermined. Our approach could readily be used to triage the vast numbers of potential competence studies into high-likelihood and high-priority combinations.

### Utility of combining animal and plant data–feature overlap

When a virus is transmitted from one host to another, either intraspecies or interspecies, and in both animals and plants, it has to persist for some length of time outside of the host. This can be for a limited amount of time and exposure for direct transmission routes, to prolonged periods of months to years for fomite and water-borne viruses [45,46].

During this time viruses are exposed to a very different environments and stressors compared to their intercellular stage, and indeed are not related to the host species/kingdom. For example, a water-borne virus compared to a fomite-borne virus will each be exposed to a more similar set of stressors to other viruses with the same transmission route–regardless of the host species or even kingdom. Therefore, we would expect some degree of homoplasy between viruses with the same transmission route, and these would be reflected in the features we use here. Looking specifically at the most common two routes found in both animals and plants, water-borne and fomite, we illustrate this.

For fomite, our data show that, overall, the 1^st^ and 4^th^ most informative viral predictors (out of 142 viral predictors, features computed on multiple ORF lengths, or multiple positions in the genome, were grouped together and their SHAP values aggregated, S3 Dataset) were proportion of positively charged amino acids and proportion of hydroxyl group containing amino acids. This was consistent across plants and animals (2^nd^ and 4^th^ for plants and 1^st^ and 4^th^ for animals, of all viral predictors), demonstrating that viruses from different kingdoms are informative to each other. Furthermore, charged and hydroxyl amino acids are well known to be important in viral adhesion to fomite surfaces [47,48], as well as aiding in thermal [49] and pH-range [50,51] stability and moisture retention [52] required by fomite-borne viruses. Icosahedral structure is also known to be prevalent in fomite-borne viruses, as it provides a rigid and compact structure able to withstand desiccation and temperature changes [53]. Icosahedral was the 5^th^ most informative viral predictor for both animals and plants.

For water-borne viruses, TT/UU nucleotide biases were the 1^st^ (out of 142, S4 Dataset) most informative viral predictors for both plant and animal water-borne viruses. High frequencies of this motif confer increased resistance to nucleases in water [54,55]. Proportion of amide and aromatic amino acids were also highly predictive (amide: 6^th^ overall viral predictor, 6^th^ plant, 6^th^ animal; aromatic 12^th^ overall viral predictor, 14^th^ plant, 10^th^ animal), and again showing remarkable similarity, and hence informativeness, across host-kingdoms. Both amino acid groups are important in protein stability, with amides forming multiple hydrogen bonds, and aromatics contribute to the hydrophobic core stability of soluble proteins, including through pi-stacking interactions [53,56,57]. These structural effects aid in aquatic thermal and pH stability [58,59].

### Limitations

We acknowledge certain methodological limitations and shortcomings in our study. Firstly, in order to synthesise meaningful features, the training of our framework has been restricted to fully sequenced viruses with at least one known animal or plant host species. While there are no theoretical limitations to deployment of our trained models, our assessment of our framework’s predictive performance cannot be extended to partially sequenced viruses. Our framework could be utilised to predict potential transmission routes of viruses without known hosts, to probable hosts, as long as diversion times are known.

Secondly, we could not integrate the full genome sequence of host species, as those data are lacking for the majority of species included. Similarly, we could not include life-history or other ecological traits of our hosts, as those data are not available for most species. Thus, we had to rely on diversion times as the only proxy to differentiate between our hosts.

Finally, our method does not make assumptions, or use features, based on which specific parts of the virus genome, or which receptor or receptor binding proteins are commonly utilised in specific transmission routes. Instead, we synthesised a wide range of features (n = 446) from three complementary perspectives. This ‘no-preconceptions’ approach enables us to analyse transmission routes/modes of viruses to their known hosts without being restricted, or biased, by our current, and highly incomplete, knowledge of the specific biological and molecular mechanisms which govern mechanism of transmission [1,2]. Whilst some of these details are known for a very limited number of well-studied viruses and hosts, they are unknown for the vast majority. Therefore, a machine learning study aiming for breadth of understanding across all transmission routes, viruses, and hosts cannot use these incomplete data. Despite this ‘no-preconceptions’ approach having this distinct advantage, it is also a limitation of the predictions, and may result in less accurate predictions for the minority of well-studied transmission routes/viruses/hosts for which important factors are well known.

## Conclusions

This study is the most taxonomically broad study of its kind, and is the first to demonstrate that viral sequence, morphology, and host information, increasingly available in the first few days of an outbreak, can be used to accurately identify the transmission routes of a novel virus, across the animal and plant viromes. Importantly, we have showcased that predictions can be achieved with high accuracy, including for respiratory and vector-borne routes/modes, which encompass the majority of high-consequence outbreaks across animals and plants. Together with the more matured field of viral host-range prediction, much of the key information which is needed to assess the potential for a virus to cause a high-consequence outbreak can be predicted in the first few days, enabling rapid and targeted mitigation procedures and triage of the time-consuming confirmatory investigative.

## Methods

### Data sources and unification

#### Viruses

We downloaded complete and reference virus sequences from GenBank [60]. Sequences without known vertebrate or plant host were excluded. Sequences labelled with the terms: ‘vector’, ‘construct’, ‘vaccine’, or ‘clone’ were also removed, as they are mainly laboratory-derived and/or manipulated. The number of ambiguous bases was identified for each sequence, and those requiring more than 1,024 permutations to resolve were excluded. Segmented viruses were included only if all corresponding individual segments met these criteria. This resulted in a total of 6,803 virus species or strains that were included in further analyses (Table A in S1 Text).

#### Virus-host associations

We compiled a comprehensive dataset of virus-host associations from relevant databases (e.g. [61–65]) and literature (e.g. [66]), mapped to virus strain/serotype (where applicable, S1 Dataset) and host species level. Identified associations were remapped to a unified taxonomy to remove any taxonomic ambiguities, and their sources were manually verified for accuracy. Associations where the underlying evidence (e.g. publication) only concurrently cite the virus and host, or specifically indicate an absence of interaction, were removed. This resulted in 28,661 associations between the above viruses and 5,750 host species (animals = 3,649, and plants = 2,101). Table B in S1 Text lists the distribution of these associations by virus Baltimore classification and host taxa. S1 Dataset lists all included associations and their sources.

#### Transmission routes

We identified 81 non-mutually-exclusive routes of virus transmission, in animals and plants, by searching relevant literature (Table C in S1 Text). The breakdown of these routes was as follows: vertical (14 routes), sexual (3), transmission via direct contact with bodily-fluids (5), feeding contact (2), direct contact (5), ingestion (3), indirect contact (2, minor routes), environmental transmission (10), arachnid-borne (2), insect-borne (28), and transmission via other-vectors (3).

We adopted a two-fold strategy to search the literature for whether our viruses are known to be transmissible (to their hosts) by one or more of our routes as follows: Firstly, we identified Title and Abstract (TIABs) of PubMed papers linked to single virus species (i.e. excluding TIABs with multiple viruses), and subsequently matched each TIAB, via keyword searches, to the transmission routes described above. The resulting routes/TIABS matches were verified, and erroneous associations removed. Secondly, we manually captured routes of transmission of viruses for which no papers were identified by the previous step, as well as for routes not detected in the TIABs, by searching through textbooks and virus sources (e.g. [66–69], S2 Dataset lists all sources).

Following a further manual check for accuracy, and to remove erroneous routes, we were able to identify at least one transmission *route* (of total = 77 routes, Table C in S1 Text) for 4,446 viruses (65.35% of total–Table D in S1 Text) to 5,317 hosts. Overall, we identified a total of 24,953 virus-host associations. S2 Dataset lists identified routes and their sources for all associations.

#### Hierarchy construction

Given a virus-host association, we considered the virus to be transmitted to the host via a parent mode (e.g. dengue virus is insect-borne to humans), if we found it to be transmissible by at least one route that is also a child node of the parent node (e.g. dengue virus is mosquito-borne to humans) in our hierarchy (Fig 1).

### Predictive features

We engineered 446 features, in three complementary perspectives, as follows (For full description see Notes 3–5 in S1 Text).

#### Viral features (Note 3 in S1 Text)

To facilitate the identification of the unique evolutionary signatures associated with specific transmission routes/modes, we synthesised 442 features from the virus genome (Table E in S1 Text). These features encompass various viral characteristics, including genome composition, Open Reading Frames (ORF) specific features, morphological properties, and replication sites.

#### Host similarity (Note 4 in S1 Text)

In order to parameterise transmission routes/modes that are closely interlinked with host taxonomy, as well as those restricted to certain taxa (e.g. plant-only or mammalian-only routes), we obtained a time tree of 4,342 plant and animal species from the Time Tree of Life [70]. We computed diversion time distance between 99.98% of all included host species pairs. These distances were used to calculate similarity between the host species of the focal virus-host association, and all other hosts, for which at least one virus is known to be transmitted by the focal route/mode.

#### Virus-host integrated neighbourhoods (Note 5 in S1 Text)

Given that closely related viruses may utilise similar transmission routes in taxonomically close hosts (e.g. majority of orthoflaviviruses are mosquito-borne in mammals and birds), as well as a diverse set of routes in taxonomically distant hosts (e.g. orthoflaviviruses may exploit sexual and vertical routes in some of their vertebrate hosts, as well as some of their arthropod vectors), we incorporated pair-wise association-level similarities in our predictive pipeline.

This was achieved by expanding the concept of phylogenetic neighbourhoods [28], so that for any given virus-host association and a transmission route/mode, we firstly identified the set of viruses most closely related to the focal virus, that are known to be transmitted to some of their hosts via the focal route/mode. We then included the hosts their viruses infect via the focal route/mode to compute three complementary features (Fig A in S1 Text):

1. *MN3H* indicates whether the focal host is susceptible, via the given route/mode, to viruses which exhibit high sequence similarity to the focal virus.
2. *MN4D* measures the average similarity between the focal association to associations between viruses in the phylogenetic neighbourhood of the focal virus, not known to be transmissible to the focal host via the given route/mode and hosts other than the focal host.
3. *MN4C* measures the average similarity of the focal association to associations between viruses in the phylogenetic neighbourhood of the focal virus, known to be transmissible to the focal host via the given route/mode, and hosts other than the focal host.

### Basic components of the predictive framework

#### Binary relevance multi-label classification

Given that the same virus species/strain may deploy multiple routes/modes in the same host species (Fig E in S1 Text), we employed a multi-label classification framework. This approach allows each virus-host association to belong to multiple routes/modes simultaneously, in contrast to traditional multi-class classification that restricts each instance to a single category. Specifically, we implemented a binary relevance [71] approach, which treats each label as an independent binary classification problem.

We adopted this approach for three reasons. Firstly, it simplifies the multi-label classification task into independent binary classifiers that can be trained and queried in parallel, making the task more scalable and computationally efficient. Secondly and importantly, it accommodates it accommodates imbalanced datasets with varying label frequencies, as is the case with our transmission routes/modes (see below). Lastly, binary relevance is highly interpretable due to the independence of predictions, which enables us to quantify and compare feature contributions for individual routes/modes.

#### LightGBM

We trained a suite of LightGBM (Lightweight Gradient Boosting Machines) [72] models per every route/mode sufficient data (n = 98, 57 routes, 41 modes, Table C in S1 Text). We elected to train LightGBM classifiers due to their efficiency in handling large-scale datasets, and ability to capture complex patterns in the data effectively. Note 7 in S1 Text provides further details of the LightGBM implementation.

### Class balancing

The proportion of observed virus-host instances varied greatly per route/mode (Figs 1 and C in S1 Text and S4 Data), ranging from 0.16% (vertical trans-egg transmission in invertebrates) to 98.71% (horizontal transmission) of the 24,953 virus-host associations with at least one observed transmission route/mode. This presented a varied and bi-directional imbalance between observed (positive class), and unknown (negative class) transmission routes/modes for our associations.

We compared the performance of 22 class-balancing resampling techniques (Table F in S1 Text lists full definitions), as well as performance of models constructed by tuning a lightGBM specific hyperparameter used to address class imbalance in binary classification tasks, across all modelled transmission routes/modes (n = 98), over a single iteration of our pipeline (see below). Performance was assessed across ten metrics measured using the corresponding held-out test-set for each route/mode (Table H in S1 Text).

**Fig 6:**
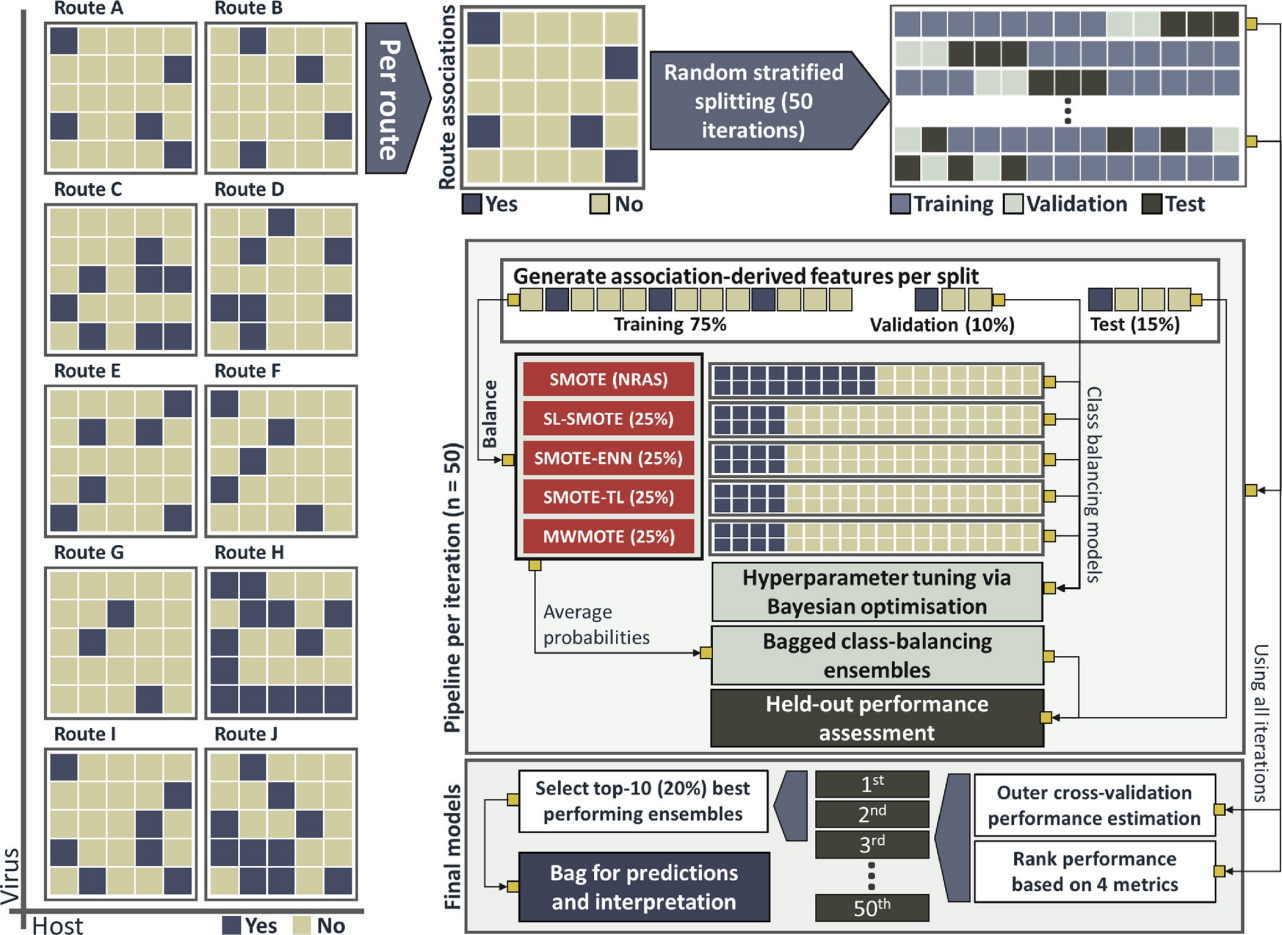
Model training, optimisation, validation, and selection. Routes/modes are treated as independent binary classifiers (n = 98). For each route, the set of virus-host associations with at least one known transmission route of virus to host (n = 24,953) is initialised such that: if the virus is known to be transmitted to the host via the focal route/mode, the association is categorised as “yes” (positive class), or else “no” (negative class). Per each iteration (n = 50 per route/mode), the initialised dataset is split into a training set (75%, blue), validation set (10%, light blue), and held-out test set (15%, red). The splits are stratified per class, so that distribution of positive/negative class in each split mirror that of the initialised dataset. In each iteration, the training set is balanced using five different class-balancing algorithms, resulting in five balanced sets. A lightGBM model is tuned, for each balanced set, using the iteration’s validation set. The probabilities resulting from the constituent five models are bagged (i.e. averaged) per iteration, and performance of resulting ensemble is assessed against the corresponding held-out test-sets. The ensembles are scored and ranked based on the average of four different metrics, and the top-10 (best 20% over the 50 iterations) are selected and used to generate final predictions and SHAP values.

No single approach outperformed all others across all metrics and all routes/modes (Fig D in S1 Text). Therefore, we incorporated five complementary class-balancing techniques into our multi-label classification framework (Fig 6), as follows: two over-sampling techniques—SL-SMOTE (25%, minority class = 25% of resulting total), and MWMOTE (25%); two over- and under-sampling hybrid techniques—SMOTE-ENN (25%), and SMOTE-TL (25%); and one over-sampling and noise reduction hybrid technique—SMOTE (NRAS, minority class = 50% of resulting total). Note 6 in S1 Text provides explanation of those techniques.

### Predictive framework workflow

#### Training, optimisation, and validation of class-balancing ensembles

In order to incorporate the uncertainty arising from the stochastic elements in mode training, as well as from the variations in class-balancing resampling techniques, we randomly cross-validated our models over 50 iterations, as follows (Fig 6):

1 –Initialisation. We first initialised an input set of virus-host associations for each of our routes/modes. Positive class comprised all associations in which the virus is transmissible to the host via the focal route/mode. Negative class comprised the remainder of our 24,953 associations with at least one observed transmission route/mode.
2 –Splitting. Per each iteration (n = 50 per route/mode), the above set was split into training (75%), validation (‘optimisation’) (10%), and held-out test (15%) sets using stratified random sampling. Validation sets were used to tune the hyperparameters of constituent models; and the test sets were used solely for performance assessment.
3 –Feature generation. Association-derived features (host similarity and virus-host integrated neighbourhoods) were recalculated for each split as follows: training (~75% of total), validation (~85% of total, including both training and validation association), and test (all data).
4 –Class balancing. We applied five class-balancing techniques to each training set, producing five balanced sets.
5 –Optimisation. we tuned a LightGBM model for each of the resulting balanced training sets using Bayesian optimisation. The optimisation process focused on two metrics: AUC and PRAUC, tuning nine hyperparameters (Table G in S1 Text). The validation set (10% of the data, not balanced, different per each iteration and route/mode combination) was used to evaluate the performance of different hyperparameter configurations and select the best set for each constituent model. Optimisation continued until no improvement was observed for 15 consecutive rounds.
6 –Bagging. We averaged the probability outputs of the five constituent lightGBM models to generate a class-balancing ensemble. This bagging approach mitigated uncertainties and improved the robustness and stability of predictions (Figs Y-AB and Tables I-J in S1 Text).
7 –Performance assessment. We evaluated the performance of the class-balancing ensembles, and their constituent models, using a comprehensive set of metrics (Table H in S1 Text) on the held-out test sets (15% of virus-host interactions per iteration, not balanced).

Steps 2–7 above were performed using the R Packages: mlr3, mlr3extralearners, mlr3tuning, mlrintermbo, and lightgbm.

#### Construction of final ensembles

We ranked the class-balancing ensembles (n = 50), trained for each route/mode (n = 98), based on the average of four metrics: (ROC-)AUC, PR-AUC, Precision, and 1-Brier score, measured using the corresponding held-out test sets. We then selected the top 10 ensembles (20%) and averaged their probabilities to generate the final predictions.

Higher values (closer to 1) of AUC, PR-AUC, and Precision indicate better performance. AUC measures the discrimination ability of binary classifiers, while PR-AUC is preferred for imbalanced datasets as it focuses on the positive class. Precision (Positive Predictive Value) further evaluates the accuracy of positive predictions. Brier score measures the accuracy of probabilities generated by the models, with lower (closer to 0) Brier scores indicating more reliable predictions. Using these complementary metrics allowed us to account for discrimination, class imbalances, positive prediction accuracy, and calibration aspects, when ranking our ensembles.

### Model interpretability

We used SHAP (SHapley Additive exPlanations) values [73] to quantify the contribution of each of our features to individual predictions. SHAP values employs cooperative game theory principles to determine the marginal impact of each feature on the difference between the actual and average predictions. SHAP values can be either positive or negative, indicating whether a feature increases or decreases the prediction probability, compared to the average prediction across all possible instances, respectively. The absolute magnitude of the SHAP value for a feature measures the importance or influence the corresponding feature has on the prediction for a specific instance. For each route/mode, we first computed local SHAP values for each constituent model (n = 50) of our top-10 ensembles, then averaged them to generate an aggregate SHAP value for each instance/feature combination.

To compare feature contributions and assess overall importance, across all modelled routes/modes, we normalised SHAP values at two levels as follows: 1) Local (locally normalised) SHAPs—quantify relative feature importance within each specific route/mode. 2) Global (Globally normalised) SHAPs—quantify relative importance across all models, which allow us to compare feature contribution across different routes/modes and identify consistent patterns of feature importance across multiple routes/modes. Additionally, we examined the stability of our SHAP values of our top-10 ensembles (Fig M in S1 Text), as well as in relation to correlation between our predictive features (Fig N in S1 Text). Figs O-R in S1 Text provide further details.

### Prediction dependencies

To maximise the utilisation of our models in mitigating against future emerging viruses, we assessed their ability to differentiate between closely related routes/modes (e.g. mosquito-borne vs midge- or sandfly-borne). To this end, we evaluated the extent to which our predictions for a given route/mode depended on predictions of its sibling route(s)/modes(s)—routes/modes that share a common parent node in our hierarchy (Fig 1). This was achieved using Mutual Information (MI) to determine the dependency between the mean probabilities (top-10 ensembles) for instances predicted to be transmitted by the focal route/mode (mean probability >0.5), and those of its siblings. For focal routes/modes with multiple siblings, we used the maximum mean probability for each instance in our calculations.

We normalised MI estimates by dividing each by the maximum possible MI given the underlying sample size. Normalised MI ranges between 0 and, where 0 indicate there is no information shared between the focal route/mode and its siblings, and therefore there is no relationship between the two; whereas a Normalised MI ≥ 0.7 suggests a strong correlation between the focal route/mode and its siblings, and changes in one are likely to be reflected in the other. Figs O-R in S1 Text provide further analyses of prediction dependencies.

### Re-predicting important secondary routes of high consequence viruses

We applied the following criteria: for each of humans, animals, and plants, we identified viruses which have had a major outbreak in the last 5–10 years; and have multiple known transmission routes, at least one of which was discovered during an outbreak. We selected the two most ‘high-consequence’ which met our selection criteria (six viruses total, excluding SARS-CoV-2).

High-consequence was defined as global health or economic burden. Viruses were selected based on these criteria from the WHO blueprint priority diseases [74] for humans (Zaire Ebolavirus and Zika virus); the WOAH list of notifiable diseases [75] for animals (Nipah Henipavirus and Porcine epidemic diarrhea virus in pigs); and, in absence of an equivalent plant virus list, from a recent review pandemics and epidemics [35] (Maize chlorotic mottle virus and Cassava brown streak virus).

We then pooled all iterations in which the corresponding virus-host associations appeared in the held-out test sets for the minor route/mode (S4 Dataset) and computed the average probability of that specific transmission route for that virus and host from the bagged probabilities of the selected iterations’ class-balancing ensembles. If the averaged probability was greater than 0.5, we classified the association as re-predicted; otherwise, we categorised it as failed to be re-predicted.

## Supporting information

S1 Dataset Viruses and virus-host associations included in this study. The dataset lists virus species or strains used in this study, their taxonomy, and identifiers, as well as all virus-host associations used and whether they were identified from sequences, peer-reviewed publications, or other sources.
(XLSX)

S2 Dataset Association-level transmission routes identified in this study. The dataset lists transmission routes, the virus-host associations they apply to (virus is transmitted to host via the given route), and sources used.
(XLSX)

S3 Dataset SHAP Values. The dataset provides mean SHAP value, variance, and spread for all features included in Fig 3, as well as mean aggregated SHAP value and standard deviation for all grouped predictors of fomite and water-borne routes.
(XLSX)

S4 Dataset Performance assessment. The dataset provides performance metrics (and their standard deviations) across the training, validation, and held-out test sets, as well as test set performance for top-10 ensembles and percentage of positive class-instances, for each route/mode. In addition, the dataset provides probabilities derived from re-predicting high-consequence viruses (6 in total).
(XLSX)

S1 Text Supplementary Text (Notes 1–7) and Supplementary Results 1–6. (PDF)
